# Circular RNAs–Stress Granule Interplay Drives Aging‐Related Proteostasis Loss

**DOI:** 10.1111/acel.70641

**Published:** 2026-07-16

**Authors:** José M. Izquierdo

**Affiliations:** ^1^ Centro de Biología Molecular Severo Ochoa (CBM) Consejo Superior de Investigaciones Científicas‐Universidad Autónoma de Madrid (CSIC‐UAM) Madrid Spain

**Keywords:** aging, CircRNAs, longevity, proteostasis, ribostasis, RNASEK, stress granules

## Abstract

Aging involves a gradual loss of cellular balance, leading to reduced function and increased disease risk. While impaired proteostasis is a key hallmark of aging, more evidence shows the importance of RNA homeostasis (ribostasis), particularly the regulation of circular RNAs (circRNAs). CircRNAs are stable RNA molecules that build up over time and are linked to age‐related cellular dysfunctions. In this regard, Kim et al. 2026 provide new insights into the impact of circRNA turnover on aging and lifespan. Their findings indicate that the accumulation of circRNAs is partly due to a decline in ribonuclease K (RNASEK), an enzyme that breaks down circRNAs. Using models such as worms, mice, and human cells, they show that RNASEK is crucial for healthy aging and longevity, suggesting its role is conserved across species. The research also shows that circRNAs gather in stress granules (SGs), which are ribonucleoprotein complexes formed during cell stress. RNASEK collaborates with heat shock protein 90 to prevent harmful RNA‐rich aggregates, maintaining cellular dynamics in balance. These findings suggest a link between ribostasis and proteostasis, identifying circRNA clearance as a potential factor in longevity. The study also points to RNASEK as a promising target for treating age‐related diseases. However, key questions remain, such as how RNASEK specifically degrades circRNAs, whether specific circRNAs or overall circRNA levels drive aging traits, and whether circRNA buildup is a cause or result of cell aging. Further research is needed to evaluate the conservation, safety, and therapeutic potential of this proteostasis‐ribostasis axis in human biology.

## Graphical abstract

Ribonuclease K (RNASEK) maintains ribostasis by degrading circular RNAs (circRNAs) in young cells, enabling functional stress granules (SG) and proper RNA‐protein metabolism for healthy aging and longevity. In aged cells, reduced RNASEK activity causes circRNA accumulation in SG, disrupting ribostasis and proteostasis, leading to cellular dysfunction, premature aging, and shorter lifespan.

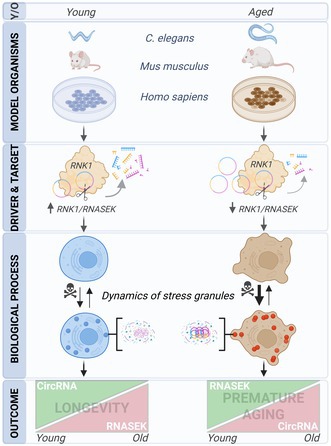

Aging is a multifactorial biological process characterized by the gradual deterioration of cellular integrity and physiological function (López‐Otín et al. 2012). A fundamental aspect of this process is the disruption of the mechanisms that maintain macromolecular homeostasis, particularly those that regulate proteins and RNA. Although the loss of proteostasis has historically been considered a primary hallmark of aging, there is growing evidence suggesting that the dysregulation of RNA metabolism plays an equally crucial role (López‐Otín et al. 2023). The convergence of these processes is particularly evident in the context of stress granules (SGs), which act as dynamic hubs for the regulation of RNA and proteins under conditions of cellular stress (López‐Otín et al. 2023; Genuth and Dillin 2025).

Proteostasis refers to the integrated network of pathways responsible for maintaining the proper synthesis, folding, transport, post‐translational modifications and degradation of proteins (Hipp et al. 2019). This network includes molecular chaperones, which assist in protein folding, as well as degradation systems such as the ubiquitin‐proteasome system and the autophagy‐lysosome pathways. Together, these systems ensure that proteins achieve and maintain their functional conformations, while preventing the accumulation of damaged or misfolded proteins (Genuth and Dillin 2025). However, with advancing age, the efficiency of these pathways declines. This loss of proteostasis leads to increased protein misfolding, aggregation, and impaired degradation, which ultimately contributes to cellular dysfunction and the onset of disease (Hartl 2016).

Along with the decline in proteostasis, RNA metabolism undergoes significant changes during aging (Aoi and Shilatifard 2023; Debès et al. 2023; Tyshkovskiy et al. 2023). Non‐coding RNAs (ncRNAs), once considered “transcriptional noise”, are now recognized as key regulators of gene expression and cellular homeostasis (Chen 2020). Among them, circRNAs have attracted considerable attention due to their unique structural and functional properties (Sanger et al. 1976). Thus, several researchers have long observed that circRNAs accumulate differentially in cells, tissues, and organs, sometimes independently organismal age, across species ranging from worms to humans (Westholm et al. 2014; Rybak‐Wolf et al. 2015; Veno et al. 2015; Dang et al. 2016; Gruner et al. 2016; Cortés‐López et al. 2018). Although they were also considered largely passive markers of aging (Zhang et al. 2016; Chen 2020). Kim et al. (2026) have recently reported that its accumulation significantly contributes to the aging process itself (Kim et al. 2026) (Figure 1).

**FIGURE 1:**
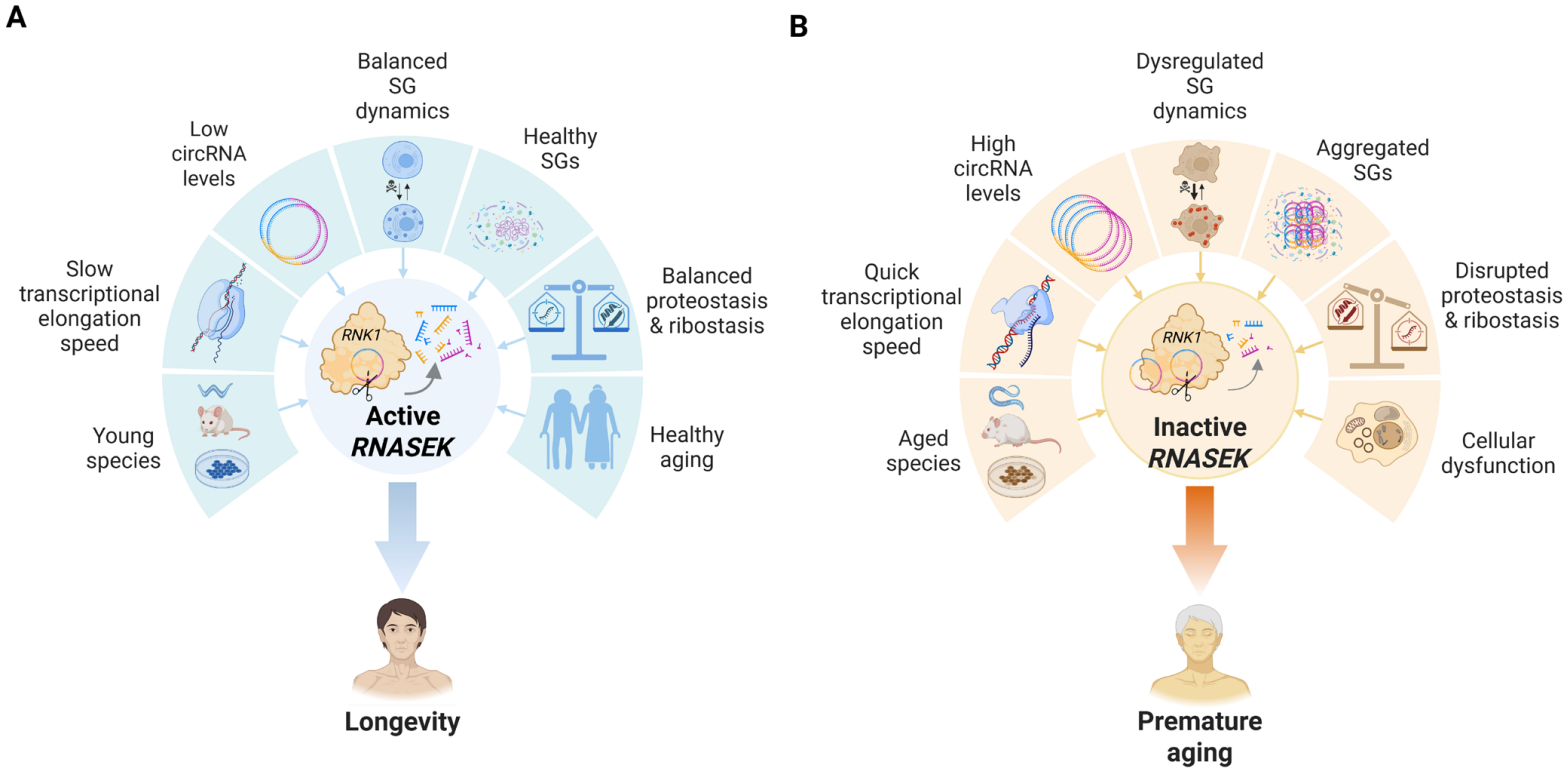
Ribonuclease K may promote longevity by preventing circular RNA accumulation in stress granules. (A) Active ribonuclease K (RNASEK), which is highly evolutionarily conserved, plays a crucial role in maintaining cellular homeostasis in young individuals. It limits the accumulation of circular RNAs (circRNAs) by facilitating their turnover, thereby preventing excessive sequestration within stress granules (SGs). This function preserves RNA homeostasis (ribostasis), supports proper SG dynamics, and maintains proteostasis by reducing the burden of aberrant ribonucleoprotein assemblies, ultimately enhancing the longevity of the organism. (B) Inactive RNASEK leads to a deleterious phenotype in aged individuals. A reduction in RNASEK activity results in the accumulation and persistence of circRNAs within SGs, contributing to degenerative SG dynamics and disrupting RNA and protein quality control systems. These changes promote a shift from adaptive stress responses to chronic cellular dysfunction, accelerating the onset of premature aging‐related phenotypes. Overall, RNASEK is identified as a key regulator at the interface of ribostasis and proteostasis, linking protein and RNA metabolism to healthspan and longevity. CircRNAs, circular RNAs; RNASEK, V‐type proton ATPase subunit F/ribonuclease K. This figure was created using BioRender.com.

In this context, they employed a specific genetic screen in the model organism *Caenorhabditis elegans* to analyze various ribonucleases. Through this analysis, they identified RNK‐1, the worm ortholog of human ribonuclease *κ* (RNASEK), as a crucial enzyme responsible for cleaving and degrading circRNAs. RNASEK is also recognized as a V‐type proton ATPase subunit F–associated protein, thereby linking it to endolysosomal function. Unlike many nucleases that cleave at the ends, RNASEK operates as an endoribonuclease, cleaving in the middle of RNA strands to initiate the degradation of circRNAs (Kim et al. 2026) (Figure 1).

The central discovery was that RNASEK expression naturally declines with age, which directly correlates with the progressive rise in circRNA levels. When RNASEK (or its RNK‐1 ortholog in *C. elegans* ) function is reduced, via genetic knockdown or mutation, circRNAs accumulate excessively. These excess circRNAs localize to stress granules (SGs), dynamic cytoplasmic assemblies that form in response to cellular stress to sequester RNAs and proteins, temporarily halting translation and preserving homeostasis. In normal conditions, SGs disassemble once stress resolves; however, in aged or RNASEK‐deficient cells, the persistent high levels of circRNAs cause SGs to become abnormally stable, prolonged, or pathologically aggregated. This disrupts RNA and protein homeostasis, impairs cellular function, and accelerates premature aging phenotypes (Kim et al. 2026) (Figure 1A,B).

The molecular mechanism involves cooperation between RNASEK, heat shock protein 90 (HSP90), and Ras‐GTPase‐activating protein‐binding protein 1 (G3BP1), which together prevent the toxic aggregation of circRNAs within SGs. Loss of RNASEK leads to unchecked circRNA buildup, toxic stress granule persistence, and downstream effects such as proteostasis collapse and faster organismal decline (Kim et al. 2026) (Figure 2).

**FIGURE 2:**
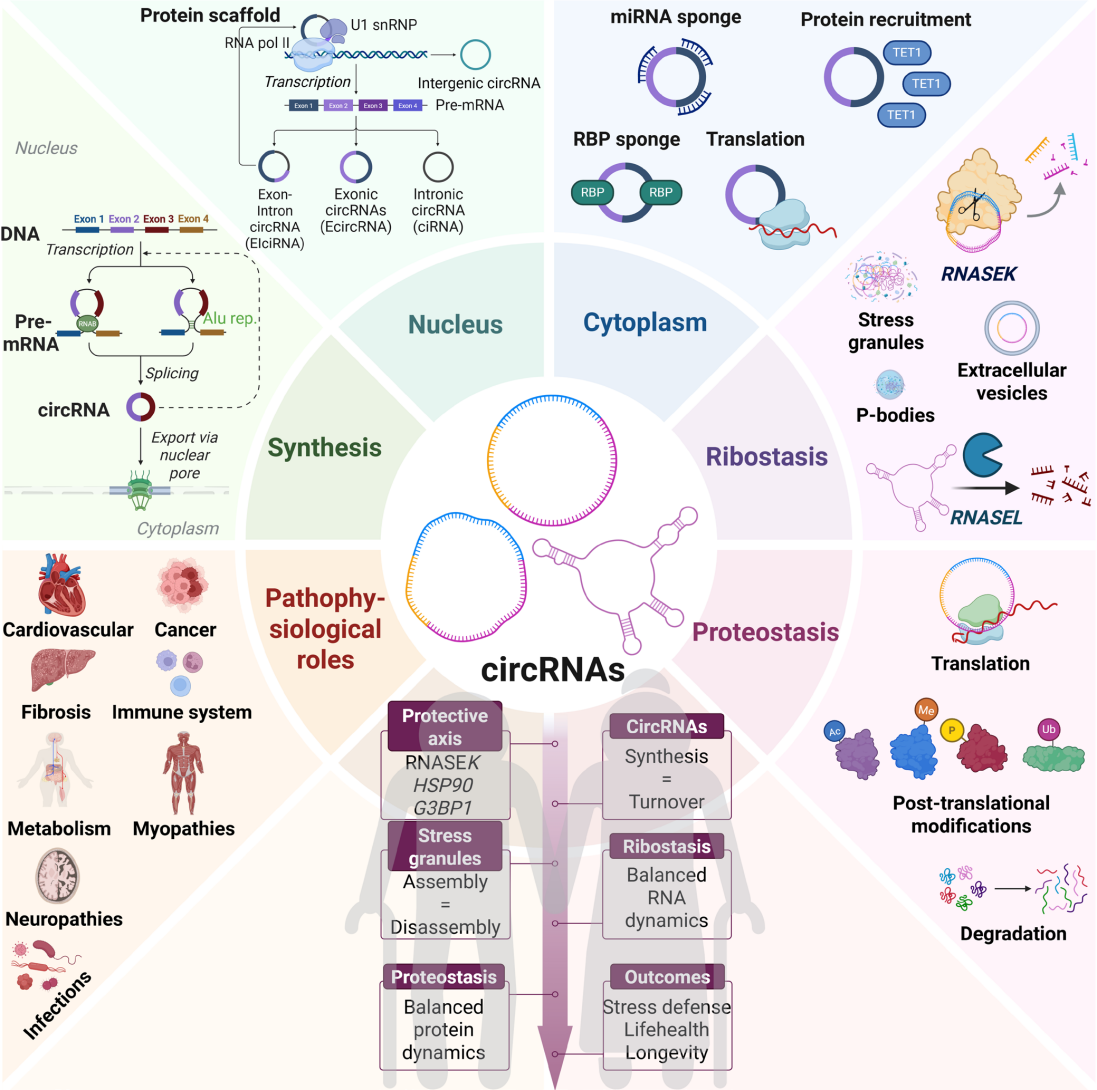
Circular RNAs as dynamic hubs with both pathological and protective functions during aging. Comprehensive overview of circular RNA (circRNAs) biology and their pathophysiological implications. Schematic representation of the synthesis mechanism of circRNAs, their nuclear and cytoplasmic functions, and their implications in ribostasis, proteostasis, and aging‐related pathophysiological processes. It schematically represents the biological lifecycle of circRNAs, beginning with their synthesis through back‐splicing within the cell nucleus. The diagrams delineate their diverse functions in both the nucleus and cytoplasm, where they actively regulate ribostasis and proteostasis, crucial for maintaining RNA and protein stability and functionality within the cell. It emphasizes how circRNAs modulate gene expression and interact with proteins and other RNA molecules, thereby influencing cellular dynamics. Furthermore, the images illustrate the diverse implications of circRNAs in various pathophysiological processes, including their involvement in cardiovascular and neurodegenerative diseases, cancer, and other pathologies associated with age dysfunctions. Finally, it highlights the contribution of circRNAs to the molecular mechanisms of aging, underscoring their potential impact on cellular homeostasis and longevity. This figure was created with Biorender.com.

Functionally, manipulating RNK‐1 levels in *C. elegans* produced striking lifespan effects: knockdown shortened lifespan by approximately 20%, while overexpression extended it by around 30%. Similar conserved roles were confirmed in mammalian models: in mice and cultured human cells, reduced RNASEK activity led to age‐associated circRNA accumulation, aberrant stress granule dynamics, and accelerated aging markers, whereas enhancing RNASEK function helped limit these detrimental changes (Kim et al. 2026) (Figure 1).

In summary, this study reconceptualizes circRNA accumulation as not merely a byproduct of aging but as an active contributor, with RNASEK identified as a conserved and evolutionarily significant regulator of longevity. By facilitating circRNA turnover and ensuring proper stress granule (SG) resolution, RNASEK enhances cellular resilience and prolongs lifespan. The authors suggest that therapeutic strategies focused on augmenting RNASEK activity, promoting circRNA degradation, or directly targeting age‐accumulated circRNAs could provide innovative approaches to address aging and age‐related diseases, including neurodegenerative disorders where SG dynamics dysregulation is prevalent. This research, encompassing genetic, biochemical, cellular, and organismal analyses from nematodes to mammals, presents compelling evidence for a novel RNA‐centric mechanism in aging biology and underscores the potential of RNA metabolism pathways as targets for interventions aimed at promoting healthy longevity (Kim et al. 2026).

While aging is traditionally characterized by a broad decline in proteostasis, another recent seminal work by Debès et al. (2023) provides critical mechanistic clarity on how RNA metabolism, specifically transcriptional elongation and circRNA biogenesis, is altered over time. Rather than a generic shift in RNA abundance, aging drives a distinct kinetic defect in transcription where the elongation rate of RNA Polymerase II (Pol II) significantly slows down (Aoi and Shilatifard 2023; Debès et al. 2023). This deceleration alters the coordination between transcription and pre‐mRNA splicing; as Pol II tracks more slowly across a gene, the window of opportunity for unconventional splicing events widens. Specifically, this kinetic delay favors back‐splicing, a process where a downstream 5′ splice site joins with an upstream 3′ splice site to form a covalently closed circular RNA molecule instead of a linear transcript (Zhang et al. 2016). Because circRNAs lack free 5′ or 3′ ends, they are inherently resistant to standard exonuclease‐mediated degradation. Consequently, the age‐associated slowdown of Pol II leads to an increased rate of circRNA biogenesis, causing these highly stable molecules to accumulate progressively in aging tissues until they breach a physiological threshold, driving concentration‐dependent circRNA aggregation (Debès et al. 2023) (Figures 1 and 2). Further, because back‐splicing is a co‐transcriptional process that exists in direct kinetic competition with canonical linear splicing, any systemic shift in transcriptional machinery will alter circRNA output. Layered upon this kinetic shift is the well‐documented phenomenon of age‐associated spliceosome decay. As cells senesce, the expression and architectural fidelity of core spliceosome components degrade (Ashwal‐Fluss et al. 2014), relaxing the structural checkpoints that normally suppress back‐splicing eventuation (Figure 2). Therefore, RNASEK clearance model is not an absolute model, unquestioned truth risks ignoring these robust transcriptional alternatives. A more holistic view suggests a multi‐hit physiological decline: an upstream elevation of circRNA biogenesis driven by transcriptional slowing and spliceosome dysregulation, which eventually overwhelms or coincides with a late‐life collapse of secondary active clear‐out pathways like RNASEK and perhaps involving additional circRNA turnover systems (Figure 2).

To fully understand this accumulation, this kinetic surge in biogenesis must be contextualized within the broader landscape of circRNA clearance (Chen 2020; Wu et al. 2022; Chen et al. 2023). While the novel endonuclease RNASEK is a crucial mediator of circRNA turnover (Kim et al. 2026), cells employ several parallel pathways to regulate circRNA levels. A primary enzymatically driven pathway involves RNase L (i.e., *RNASEL* gene), which degrades structured circRNAs upon viral infection or generic immune activation, operating as a critical checkpoint for innate immune signaling. Also, circRNAs are targeted through global RNA silencing machinery, such as the cytoplasmic Ago2‐mediated miRNA endonuclease pathway, which cleaves specific circRNAs containing perfectly complementary miRNA binding sites into P‐bodies (Chen et al. 2023; Beadle et al. 2023). In the same vein, structured circRNAs can also undergo endonucleolytic cleavage by the dynamic interactions of the as G3BP1‐ regulator of nonsense transcripts 1 (UPF1) complex during ordinary RNA surveillance (Boo et al. 2024). Further, physical clearance mechanisms complement these enzymatic pathways. When intracellular degradation capacity becomes saturated, as often observed during advanced aging, cells actively export circRNAs via extracellular vesicles (EVs), such as exosomes, to maintain intracellular homeostasis (Chen 2020; Chen et al. 2025).

Together, the progressive deceleration of Pol II elongation, combined with the eventual saturation or decline of these multi‐layered degradation pathways, perhaps explains the distinct accumulation and aggregation of circRNAs during aging. This exceptional dual‐kinetic model allows circRNAs to build up over time, particularly in long‐lived, postmitotic cells like neurons (Pan et al. 2020; Wu et al. 2022; Nemeth et al. 2024), resulting in a significantly longer half‐life compared to their linear counterparts (Wu et al. 2020; Wu et al. 2022). However, circRNAs can also be accumulated during pre‐adult and embryonic development stages (Dang et al. 2016; Beadle et al. 2023). These early‐stage accumulation challenges age‐dependent increase behavior (Kim et al. 2026). Perhaps different adaptation mechanisms are operating associated with heterogeneous circRNAs population synthesis and/or degradation timing during cell/tissue/organ‐specific reprogramming and diversity along development from birth to late adult age.

In this regard, an extensive single‐cell landscape of circRNAs by utilizing large‐scale, full‐length single‐cell RNA sequencing (scRNA‐seq) across diverse tissues, developmental stages, and cellular states. By analyzing back‐splicing junction sequences from primarily exonic regions, the researchers successfully identified 139,643 circRNAs in human single cells and 214,747 in mouse single cells. A key finding of the research is that circRNAs are significantly enriched in neurons compared to other brain cell types, with inhibitory and excitatory neurons exhibiting distinct, cell‐specific expression patterns that correlate with RNA‐binding protein (RBP) levels. This highlights a highly specific, RBP‐regulated expression mechanism across different cell types. Furthermore, the study revealed substantial cellular heterogeneity, identifying 12,625 circRNAs unique to a single cell type, which underscores the necessity of single‐cell level investigations to fully understand the role of circRNAs in tumor development. By generating a circRNA reference for eight immune cell types, the researchers demonstrated that using circRNAs as cell‐type signatures yields greater accuracy in cell composition deconvolution than traditional gene markers alone, establishing circRNAs as promising biomarkers for profiling tumor‐infiltrating immune cells. Thus, the study emphasizes the highly specific expression of circRNAs at an unprecedented resolution, pointing to the critical need for developing advanced single‐cell and spatial sequencing technologies tailored for circRNA detection (Wu et al. 2022) (Figure 2).

CircRNAs perform various regulatory functions, including acting as transcriptional regulators, microRNA sponges, interacting with RNA‐binding proteins, and modulating transcriptional and translational processes (Chen 2020) (Figure 2). While these functions may contribute to cellular resilience under certain conditions, the progressive accumulation of circRNAs during aging raises important questions about their long‐term impact on cellular homeostasis and disease (Pan et al. 2020; Wu et al. 2020; Wu et al. 2022; Nemeth et al. 2024). In particular, their persistence and resistance to degradation suggest that they may influence the dynamics of transient RNA‐protein assemblies, such as SGs and cell signaling pathways (Kim et al. 2026) (Figure 2).

The physiological impact of circRNAs during aging is further complicated by their subcellular compartmentalization and non‐canonical translational capabilities, both of which introduce distinct variables into the SG interaction model. For instance, within the complex geometry of the neuron, circRNAs are highly enriched within synaptic fractions and dendritic spines (Rybak‐Wolf et al. 2015). Synaptic terminals operate as autonomous metabolic zones requiring localized activity‐dependent translation. This profound synaptic pooling raises critical questions regarding local ribostasis. Synapses are exceptionally sensitive to localized oxidative and metabolic stresses, triggering the formation of micro‐compartmentalized, synaptic SGs that temporarily sequester translation machinery. Thus, an age‐associated decline in local synaptic clearance mechanisms (i.e., synaptic RNASEK activity) would lead to the persistent and irreversible stability of local SGs. This localized ribostasis failure would disrupt synaptic proteostasis and local protein synthesis, inducing dendritic spine collapse and synaptic pruning long before systemic somatic death occurs.

Furthermore, our understanding of circRNAs must accommodate the definitive evidence that a subset of these molecules, potentially ncRNAs, is translationally active. Driven by internal ribosome entry sites (IRES) or N6‐methyladenosine (m6A) modifications, certain circRNAs undergo cap‐independent translation (Yang et al. 2017; Wu et al. 2023; Margvelani et al. 2025). This coding capacity has immediate, severe implications for neurodegenerative diseases characterized by nucleotide repeat expansions, such as C9orf72‐associated amyotrophic lateral sclerosis and frontotemporal lobar degeneration. In these contexts, unconventional back‐splicing generates translation‐competent circRNAs that undergo rolling‐circle translation, yielding toxic dipeptide repeat proteins that seed intracellular aggregation (Ashwal‐Fluss et al. 2014).

When integrated with the SG paradigm, a pathogenic feed‐forward loop emerges. SGs containing untranslated circRNAs may not merely act as passive physical blocks; if these sequestered, translation‐competent circRNAs escape timely endonucleolytic clearance, they may undergo aberrant translation directly within or adjacent to the granule matrix. The resulting proteotoxic peptides would accelerate the liquid‐to‐solid phase transition of SGs into pathological and permanent aggregates linking RNA decay failures directly to proteinopathic decay. Thus, circRNAs are not merely passive waste products whose accumulation marks the passage of time; they can serve as active upstream genetic modulators of organismal longevity. In fact, functional genetic screens in *Drosophila melanogaster* have revealed that the targeted ablation or overexpression of specific circular loci (such as *circSuji* or *circMbl*) can significantly extend or shorten lifespan and preserve locomotor function during aging (Weigelt et al. 2020). These lifespan extensions occur independently of changes to corresponding linear host transcripts, indicating that individual circRNAs exert deliberate regulatory control over systemic metabolic and proteostatic signaling networks. Therefore, a breakdown in active clear‐out systems like the RNASEK pathway does not simply cause physical crowding of the cytoplasm. It actively scrambles the precise, homeostatic circRNA networks evolved to delay neuromuscular and cognitive decline, offering a deeper molecular bridge between RNA metabolism, SG dynamics, and the regulation of healthspan.

SGs are membrane‐less organelles that form rapidly in response to environmental stressors, such as oxidative stress, heat shock, viral infections, or nutrient deprivation. They arise through liquid–liquid phase separation (LLPS) and are composed of various types of RNA, including linear RNAs (both short and long), non‐coding RNAs (ncRNAs), circRNAs, and coding RNAs (such as translationally stalled mRNAs), as well as RNA‐binding proteins (RBPs) and components of the translational apparatus, among others (Protter and Parker 2016; Chen et al. 2023). Studies investigating the composition of SGs have shown that they contain more than 450 proteins and over 11,000 transcripts (https://rnagranuledb.lunenfeld.ca). Functionally, SGs serve to temporarily halt protein synthesis, protect mRNAs from degradation, and prioritize the translation of housekeeping and stress‐response genes as an adaptive survival response to prevent cell death. By reducing the burden on the protein folding and synthesis machinery, SGs play an important role in maintaining ribostasis and proteostasis under conditions of acute stress (Protter and Parker 2016; Hofmann et al. 2021).

However, the dynamics of SGs are strictly regulated, and their persistence beyond the period of stress can have harmful consequences. In young and healthy cells, SGs are transient structures that disintegrate once normal conditions are restored (Figure 1B). In contrast, aging is associated with defects in the clearance of SGs, leading to their persistence and, in some cases, their transition into more stable and solid‐like aggregates (Figure 1B). These abnormal accumulations can sequester essential proteins and RNAs, disrupt cellular processes, and contribute to the pathogenesis of age‐related diseases (Kim et al. 2026) (Figures 1 and 2).

The interaction between circRNAs and SGs represents a key point of convergence between RNA metabolism and proteostasis. Under stress conditions, circRNAs are recruited to SGs alongside linear RNAs, RBPs, and other cellular proteins. Due to their circular structure and resistance to degradation, can, in principle, persist within these assemblies for long periods of time. This persistence could allow them to act as scaffolds that stabilize SGs, influencing their size, composition, and material properties. As circRNAs accumulate, their growing presence within SGs could contribute to reduced dynamics and increased stability of these structures, reprogramming the proteome and transcriptome, and affecting cellular ribostasis and proteostasis (Kim et al. 2026) (Figures 1 and 2).

Nevertheless, recent advancements have considerably enhanced our comprehension of ncRNAs as structural and regulatory frameworks within biomolecular condensates (reviewed by Chen et al. 2026). Foundational research has demonstrated that ncRNAs are prevalent constituents of membraneless organelles, contributing to both SGs, P‐bodies, and other RNA granules (Chen et al. 2026). ncRNAs are particularly adept at facilitating LLPS. They function as multivalent platforms that promote the recruitment and partitioning of specific RBPs and complementary linear transcripts into these dense cellular compartments. By augmenting the local concentration of macromolecular components, ncRNAs (and also circRNAs) effectively reduce the thermodynamic barrier necessary for phase separation, thereby influencing the assembly, stability, and disassembly of stress‐responsive condensates (Wu et al. 2020; Wu et al. 2022; Chen et al. 2023). Thus, circRNAs of less than 600 nucleotides in length with a higher capability of binding SG‐related RBPs show a stronger preference to be recruited into SGs (Wu et al. 2020; Chen et al. 2023). However, certain small ncRNAs can also suppress SG formation (Chen et al. 2023; Chen et al. 2026).

Kim et al. (2026) challenge the existing paradigm by linking circRNA‐mediated LLPS directly to the pathophysiology of aging. In young cells, physiological LLPS is a dynamic and reversible process that recruits proteins and RNAs into functional condensates. However, aging disrupts this homeostatic balance. As the age‐associated slowing of transcriptional elongation causes circRNAs to accumulate, perhaps their concentration and length eventually surpass a critical threshold. This triggers a transition from functional LLPS to irreversible and pathological macromolecular aggregation. By bridging the gap between RNA scaffolding in membraneless organelles and the chronic accumulation of circRNAs, this study uncovers previously unknown kinetic and structural mechanisms underlying cellular decline. Nevertheless, further research is required to fully elucidate these specific pathways.

A key caveat when evaluating novel models of circRNA homeostasis, such as the active clearance pathway mediated by RNASEK described by Kim et al. (2026), is the highly divergent landscape of circRNA dynamics across different biological contexts. Treating age‐dependent circRNA accumulation as a uniform and systemic hallmark of mammalian senescence misrepresents a fundamental property of these molecules: their profound tissue specificity. Comparative transcriptomics across diverse animal models have robustly demonstrated that the age‐dependent accumulation of circRNAs is heavily polarized toward the central nervous system. Initial discoveries in flies, worms, mice, pigs, and humans (Westholm et al. 2014; Cortés‐López et al. 2018; Hanan et al. 2017; Weigelt et al. 2020) established that circRNAs track with chronological age predominantly within neural lineages. This spatial restriction is equally pronounced in mammalian systems; cross‐sectional analyses of aging mice and human post‐mortem cohorts confirm that while peripheral organs exhibit static or fluctuating circRNA profiles, the aging brain, particularly the cortex and hippocampus, displays a progressive, dramatic upregulation of circular transcripts (Gruner et al. 2016; Kirio et al. 2025).

The mechanistic underpinning of this neuro‐specificity is intimately tied to cellular turnover and the intrinsic biophysical properties of circRNAs. Lacking free 5′ and 3′ termini, circRNAs are natively immune to standard exonucleolytic degradation pathways, displaying intracellular half‐lives that vastly exceed those of their linear counterparts (Jeck et al. 2013). In rapidly dividing peripheral tissues, circular transcripts undergo continuous dilution through successive rounds of mitosis. Conversely, long‐lived, post‐mitotic cells like neurons lack this cellular dilution mechanism.

Crucially, this accumulation is not exclusively a feature of late‐life senescence. High‐throughput sequencing reveals that circRNAs undergo an upward trajectory beginning as early as embryonic development and continuing steadily through pre‐adult maturation (Rybak‐Wolf et al. 2015; Veno et al. 2015). Consequently, the temporal profile of circRNAs in the central nervous system may represent the mathematical inevitability of a lifelong passive sink, rather than a sudden, broad failure of active degradation machineries in the twilight of organismal life. While Kim et al. employ in vitro human cell lines to dissect an active RNASEK‐driven decay mechanism under acute stress, these findings must be interpreted cautiously when translated in vivo, where peripheral clearance mechanisms remain competent or baseline back‐splicing is tightly repressed outside the neural niche. Therefore, a central theme emerging from these observations is the organismal balance between synthesis *de novo*, molecular stability and turnover. While synthesis and turnover are essential for preserving functional molecules, excessive stability, such as that exhibited by circRNAs, can become problematic if not counterbalanced by effective degradation mechanisms. Aging appears to disrupt this balance, leading to the production and accumulation of both proteins and RNA species that are resistant to turnover. Restoring this balance may be a key strategy for promoting healthy aging and longevity (Izquierdo 2023; Chen et al. 2025) (Figure 2).

Despite significant progress, many questions remain unanswered. The mechanisms underlying circRNA synthesis, degradation, and/or splicing‐mediated control are not fully understood, and it is unclear how these processes are regulated across different organisms, organs, tissues, and cells during aging. Similarly, the molecular determinants governing SGs dynamics and their transition into pathological aggregates require further investigation. It also remains unclear how universal this mechanism is. The interactions among RNASEK, other degradation systems, circRNAs, and protein quality control processes have not yet been fully elucidated, particularly in the context of RNA‐protein co‐aggregation in age‐related chronic diseases (e.g., cardiovascular and metabolic disorders, cancer, fibrotic and immunological dysfunctions, muscular and neurodegenerative diseases, dysbiosis, etc.) (Figure 2).

Future research should address these gaps by integrating multidisciplinary approaches from molecular biology, biophysics, and systems biology. In particular, elucidating the precise subcellular localization of RNASEK, its role and crosstalk in the degradation of circRNAs (and potentially linear RNAs) with other cellular turnover systems, and how it can be therapeutically targeted to promote healthy aging will be essential. Equally important is understanding how the circRNAs heterogeneity and/or translational behavior influence LLPS, how SG dynamics are regulated, and how these processes intersect with proteostasis and ribostasis. Such insights will be critical for developing novel therapeutic strategies. Targeting RNA metabolism, enhancing specific degradation pathways, and modulating SG dynamics represent promising avenues for intervention in age‐related diseases (O'Leary et al. 2025). In fact, despite their vital role as essential facilitators of key cellular functions, circRNAs were originally misclassified as non‐coding molecules due to rigid and traditional structural definitions. While this field of research is still in its infancy, recent studies have successfully demonstrated that these circular molecules actually play critical roles in the development and progression of human malignancies. Unlocking the translation mechanisms of circRNAs promises to uncover a previously hidden segment of the human (micro)proteome, offering a far deeper understanding of their significance in aging‐related diseases, a domain that has remained largely unexplored until now. Evidence reveals that certain circRNAs execute their biological activities through a dual approach, functioning both by translating into functional peptides and by acting through RNA‐based regulatory mechanisms. Furthermore, the direct physical interactions between circRNAs and proteins can alter protein modifications, subsequently steering various vital cellular pathways. This multifaceted functionality has ultimately inspired the development of innovative clinical strategies designed to manage and treat diseases by specifically targeting these unique circRNA‐protein interactions (O'Leary et al. 2025; Margvelani et al. 2025).

In summary, aging can be viewed as a progressive inability to maintain ribostasis and proteostasis, both within cells, tissues and organs, and across species (Tyshkovskiy et al. 2023; Wu et al. 2020; Wu et al. 2023; Debès et al. 2023; Kim et al. 2026). The loss of proteostasis, coupled with the accumulation of circRNAs (and their potential products) in the absence or downregulation of RNASEK and other turnover systems, may converges in SGs, leading to the formation of persistent RNA‐protein assemblies that disrupt cellular function and signaling pathways (Zhang et al. 2020). Maintaining a balance between synthesis, stability and turnover is potentially essential for preserving organ‐specific and age‐specific cellular integrity, as well as for promoting rejuvenation and longevity (Schaum et al. 2020; Zhang et al. 2020). As we advance our understanding of these interconnected processes, we may discover new strategies to extend healthy lifespan and mitigate age‐related chronic diseases (Figure 2).

## Author Contributions

J.M.I. conceptualized and wrote the commentary.

## Funding

This work was supported by Ministerio de Ciencia, Innovación y Universidades, PID2024‐160994OB‐I00.

## Conflicts of Interest

The author declares no conflicts of interest.

## Data Availability

Data sharing not applicable to this article as no datasets were generated or analysed during the current study.
